# Too hot to thrive: a qualitative inquiry of community perspectives on the effect of high ambient temperature on postpartum women and neonates in Kilifi, Kenya

**DOI:** 10.1186/s12887-023-04517-w

**Published:** 2024-01-13

**Authors:** Adelaide Lusambili, Sari Kovats, Britt Nakstad, Veronique Filippi, Peter Khaemba, Nathalie Roos, Cherie Part, Stanley Luchters, Matthew Chersich, Jeremy Hess, Kadidiatou Kadio, Fiona Scorgie

**Affiliations:** 1 Present Address: Environmental Health and Governance Center, Leadership and Governance Hub, School of Business, Africa International University, Nairobi, Kenya; 2 Centre on Climate Change and Planetary Health, London School of Hygiene & Tropical Medicine, London, UK; 3 Division of Pediatric and Adolescent Medicine, Institute of Clinical Medicine, University of Oslo, Oslo, Norway; 4 Faculty of Epidemiology and Population Health, London School of Hygiene & Tropical Medicine, London, UK; 5 Institute for Human Development, Aga Khan University, Nairobi, Kenya; 6 Department of Medicine, Clinical Epidemiology Division, Karolinska Institutet, Stockholm, Sweden; 7 Department of Public Health, Environments and Society, London School of Hygiene & Tropical Medicine, London, UK; 8 Department of International Public Health, Liverpool School of Tropical Medicine (LSTM), Liverpool, UK; 9 Wits Reproductive Health and HIV Institute (Wits RHI), Faculty of Health Sciences, University of the Witwatersrand, Johannesburg, South Africa; 10 Emergency Medicine, Env & Occ Health Sciences, and Global Health, University of Washington, Washington, USA; 11 Institute de Recherche en Siences de la Santé, Bobo-Dioulasso, Burkina Faso

**Keywords:** Heat, Neonates, Kenya, Exclusive breastfeeding, Climate change, Maternal health, Infant care

## Abstract

**Objective:**

To understand community perspectives on the effects of high ambient temperature on the health and wellbeing of neonates, and impacts on post-partum women and infant care in Kilifi.

**Design:**

Qualitative study using key informant interviews, in-depth interviews and focus group discussions with pregnant and postpartum women (*n* = 22), mothers-in-law (*n* = 19), male spouses (*n* = 20), community health volunteers (CHVs) (*n* = 22) and stakeholders from health and government ministries (*n* = 16).

**Settings:**

We conducted our research in Kilifi County in Kenya’s Coast Province. The area is largely rural and during summer, air temperatures can reach 37˚C and rarely go below 23˚C.

**Data analysis:**

Data were analyzed in NVivo 12, using both inductive and deductive approaches.

**Results:**

High ambient temperature is perceived by community members to have direct and indirect health pathways in pregnancy and postpartum periods, including on the neonates. The direct impacts include injuries on the neonate’s skin and in the mouth, leading to discomfort and affecting breastfeeding and sleeping. Participants described babies as “having no peace”. Heat effects were perceived to be amplified by indoor air pollution and heat from indoor cooking fires. Community members believed that exclusive breastfeeding was not practical in conditions of extreme heat because it lowered breast milk production, which was, in turn, linked to a low scarcity of food and time spend by mothers away from their neonates performing household chores. Kangaroo Mother Care (KMC) was also negatively affected. Participants reported that postpartum women took longer to heal in the heat, were exhausted most of the time and tended not to attend postnatal care.

**Conclusions:**

High ambient temperatures affect postpartum women and their neonates through direct and indirect pathways. Discomfort makes it difficult for the mother to care for the baby. Multi-sectoral policies and programs are required to mitigate the negative impacts of high ambient temperatures on maternal and neonatal health in rural Kilifi and similar settings.

**Supplementary Information:**

The online version contains supplementary material available at 10.1186/s12887-023-04517-w.

## Introduction

In recent decades, Africa has witnessed a surge in the frequency and duration of heat waves associated with climate change [1]. In Kenya, this increase is notable in dry and arid areas of the East Coast and Eastern parts of Turkana, Garissa, Isiolo, Mandera and Marsabit counties [2]. Kenya has also been experiencing significant drought events, exacerbating food and water security challenges across numerous communities and precipitating severe hardships. This is particularly pronounced among vulnerable groups, such as pregnant women, children and newborns, rendering the intersection of environment, climate and health a critical focal point for research [3, 4].

Epidemiological and physiological studies have shown that neonates (babies under 28 days) are very susceptible to extreme temperatures. Their limited ability to thermoregulate and dependence on caregivers underscores their vulnerability [5–7]. High ambient temperatures may affect neonatal health through four interconnected pathways: infectious diseases, nutrition and hydration, ability to care for the newborn and related health systems [8–11]. In particular, high temperatures may increase the risk of morbidity and mortality associated with infectious diseases, such as diarrheal diseases and malaria, among infants in low income settings. In addition, under high temperatures and challenging environmental and farming conditions, maintaining optimal breastfeeding practices may become a challenge as mothers require enough nutritious food and fluids to help in breastmilk production [12]. A systematic review by Edney et al. found that breastfeeding is sufficient for infants’ hydration needs even in hot weather [13]. Moreover, breastfeeding practices vary seasonally and may be influenced by factors such as agricultural activities. In Ethiopia, a study examining the time women spend planting and harvesting versus time spend engaging in exclusive breastfeeding found that women were unlikely to breastfeed when rainfall was higher (25 cm of average monthly rain versus 5 cm). This is because they spend most of their time farming, were unlikely to attend antenatal clinics, and are less likely to receive information on optimal breastfeeding behaviors [14]. In a study of infant feeding practices in Burkina Faso, we found an effect of high temperature after adjusting for the effects of the season (a 23-minute reduction in daily breastfeeding duration per 10 °C increase in temperature) [15].

Finally, high temperatures are associated with adverse effects on maternal mental health and wellbeing. High temperatures are associated with increased anxiety [16], lack of sleep [17, 18] and irritability [19], which might have a repercussion on mothers and how they take care of their Infants. Recognizing the interconnectedness of maternal and neonatal welfare is imperative, especially in regions with limited research on the effects of high temperatures on mothers and their neonates.

In sub-Saharan Africa, there is limited research on the effects of high ambient temperatures on mothers and their neonates. In addressing the research gap, our study in Kilifi County, Kenya, explores the community’s observations of the effects of high ambient temperatures on women during pregnancy, postpartum, and their neonates. As part of the CHAMNHA [Climate, Heat Maternal and Neonatal Health Africa] transdisciplinary research project, our findings contribute valuable insights into understanding the complex interplay between climate, heat, maternal health, and neonatal outcomes in the region, offering critical information for policy and intervention strategies. Previously, we have found that pregnant women are negatively affected by heat, and it interferes with their daily activities [20]. This paper focuses on the observed effect of high ambient temperatures on postpartum.

## Methods

### Research design

A qualitative descriptive study involving focus group discussions (FGDs), in-depth interviews (IDIs) complemented by key informant interviews (KIIs). The research was conducted between January 2021 to April 2022, the hottest months in Kilifi [21] (temperatures are described in Table 1). We used the Consolidated Criteria for Reporting Qualitative Research (COREQ): a 32-item checklist for interviews and focus groups (appendix 2) [22].

### Research context

This study was conducted in Kilifi County in Kenya’s Coast Province, in the sub-counties of Kaloleni and Rabai, which have a total population of 304,778. Kilifi County has an annual crude birth rate of 26 per 1,000 population [23, 24]. According to the Kilifi 2022 factsheet, literacy levels in the region are relatively low, with only 51% of males and 49% of females having successfully completed primary education. The predominant economic activity in the region is tourism. From a socio-economic standpoint, the sub-counties rank among the most impoverished constituencies along the coastal region, with 70% of the population residing below the poverty line, in contrast to the national rate of 47% [25]. Maternal, neonatal, and child health indicators in the county fall below the national average [25, 26]. Kilifi County exhibits one of the highest under-5 mortality rates in Kenya, with 87 deaths per 1,000 live births, despite the government’s initiative to offer cost-free facility-based antenatal, delivery, and postpartum services to all women [27]. Given its rural nature, communities are widely dispersed geographically, and health facilities are often situated at a considerable distance from these communities [28, 29]. Homesteads are large, comprising grandchildren, co-wives, parents-in-law, and other older relatives. During the late stages of pregnancy and childbirth, some women receive support from their mothers-in-law, who assist with daily living activities.

### Study population and sampling

We conducted interviews with 99 participants across Kaloleni and Rabai sub-counties, Kilifi, involving 10 in-depth interviews with pregnant women and 12 with postpartum mothers. Additionally, nine focus group discussions (FGDs) were organized, with three each for mothers-in-law, male spouses, and Community Health Volunteers (CHV). Sixteen key informant interviews were conducted, representing diverse sectors such as health, water, agriculture, meteorology, local administration, public health, religious institutions, environmental advocacy, and traditional birth attendants (TBAs). This comprehensive sampling strategy helped elicit a heterogeneity of perspectives, supporting robust triangulation of participant narratives. For instance, engagement with pregnant and breastfeeding women, their close family members, and CHVs guiding them through the delivery process provided firsthand insights into their lived experiences.

### Recruitment and inclusion and exclusion criteria

Aga Khan University has worked in Kilifi for the past six years and has built successful social networks with CHVs and facility health workers. We used these networks to recruit study participants in rural areas of Kilifi.

Participants were eligible for the IDIs if they were willing and able to provide informed consent and audio recorded during the interview. Pregnant women were eligible for recruitment if their pregnancy were at least 28-week gestational age as confirmed by a healthcare worker. Both primi- or multi-gravidae were eligible to participate in the study. Postpartum women were eligible at 4 to 8 weeks after childbirth. We considered the ethical implications of the additional physical and mental burden of interviewing new mothers immediately after birth in the heat. We wanted mothers to be comfortable enough to narrate their experiences. Although we intended to interview mothers up to 8 weeks, all the mothers we interviewed had babies between 4 and 5 weeks. Key informants were recruited if they held a key leadership role in the community or in relevant county ministries such as Health, Water, Agriculture, and Meteorology in the past two years.

Based on the inclusion criteria, facility health workers and CHVs identified potential interviewees through the health facilities and in the communities. To reduce bias, all identified interviewees were further screened by the research team to ensure that they made the criteria, were willing to consent and participate in the study, and their male spouses/partners had been informed. Following recruitment, interviews were scheduled at an appropriate time and date. Participants were screened by the field researchers prior to interviews to ensure that they met all the inclusion criteria. This was the first study on climate change heat exposure’s impact on maternal health, and the women we interviewed had not been interrogated on this topic.

### Data collection

The research was designed by medical anthropologists (AL and FS) and reviewed by CHAMNHA team members with extensive research design experience. AL, a Kenyan social scientist, supervised field data implementation. Field researchers had extensive experience conducting public health and community research in Kilifi and received hands-on training that included role-playing using the study guide and feedback sessions.

The data collection tools were piloted in one FGD with CHVs, a pregnant woman, and a postpartum woman. This revealed that some terms used in the interview guides were likely to be misunderstood. For instance, the term ‘high ambient temperatures’ was sometimes confused with ‘fire’ or ‘drought’, and the word ‘facilities’ was assumed to mean ‘accommodation’. The field team discussed appropriate terminology to reduce confusion (for example, adding the word ‘health’ before facilities and using the local terms *“Joto”* or *“Jua kali”* (translated as “when it is extremely hot”).

### Interviewing

Individual In-Depth Interviews (IDIs) and FGDs were conducted by researchers AL, PK, and SC at the nearest health facilities to participants’ homes, either within a designated room or outdoors beneath a tree. Each FGD comprised six to nine participants. Informed consent procedures were initiated at the commencement of the FGD, during which the researchers delivered comprehensive information in Kiswahili about the study at the group level. Subsequently, consent for participation and permission to individually audio record were obtained from each participant. Those unable to provide a written signature were assisted to consent using a thumbprint.

Interviews for key informants were conducted in their offices, which they considered most convenient. These interviews were conducted in both English and Kiswahili, as most of the participants in this group could read and write in English. An open-ended, flexible topic guide (appendix 3) was used to structure the discussions to encourage free-flowing dialogue. The IDIs and KIIs took at most one hour, while FGDs took one to two and a half hours. The study guide had broad introductory questions and explored themes on both the direct and indirect effects of high temperatures on pregnant and post-partum women. Themes explored include indigenous knowledge and beliefs about how high ambient temperatures affect pregnant and postpartum women, the developing foetus and the neonate; local norms around strategies and practices to manage high ambient temperatures during pregnancy and in the postpartum period; the role of men and other family members supporting pregnant and postpartum women and their neonates; existing interventions and actions in the community to help vulnerable populations when it is very hot, and the usefulness of daily or seasonal climate forecasts. Snacks and drinks were provided at the venue for all the participants, and transport costs for travelling to the venue were reimbursed. All participants were informed of COVID-19 preventive measures and the field team provided hand sanitizer and face masks.

### Data saturation

Guest et al. [30] note that in qualitative research, data saturation occurs within the first twelve interviews. In this study, we considered sample sizes for IDIs, FGDs and KIIs adequate for answering study questions and as reflecting the point at which data saturation was reached. We started with in-depth interviews, which were accompanied by detailed field notes. These field notes from IDIs were shared and deliberated upon daily among all researchers throughout the data collection process. Key themes, such as the indirect and direct effects of heat on both the mother and the baby, were identified and further explored in subsequent FGDs and KIIs until saturation was attained.

### Data management and analysis

Data transcription was done verbatim by a professional transcriber, assisted by study researchers. All field audio recordings, transcribed data and field notes were saved on the Aga Khan University’s data-protected password computer. For transcription accuracy, two researchers (PK and AL) checked the transcripts against the audio recordings. We followed Braun and Clarke’s (2006) thematic analysis framework to guide the analysis [31]. Data were analysed using NVIVO 12 software.

#### Data familiarization

During data collection, study researchers (AL, PK and FS) read all the field notes and discussed the key issues as they emerged in the data collection process.

#### Generating initial codes

A codebook was developed following open coding of a small sample of transcripts, using both deductive (topics that were derived from the interview guides) and inductive (findings emerging from interviewees’ narratives) analysis. Using both deductive and inductive approaches allowed us to have a more nuanced understanding of participants’ experiences of caring for neonates in extreme heat situations.

#### Searching for themes (categories)

AL examined data on the impact of high temperatures on postpartum women and their neonates by extracting and applying segments of the texts across the IDIs, FGDs and KIIs. This process entailed reviewing coding outputs and comparing them to identify salient patterns and themes for data interpretation. Categories were then charted manually in a matrix to help visualize possible relationships between themes, as illustrated in Fig. 1.

#### Reviewing themes

The data were checked and discussed with members of the larger team, to ensure saturation had been reached on the themes as presented below.

## Results

### Participants’ socio-demographic characteristics

We interviewed 12 postpartum mothers, 10 pregnant women, 19 mothers-in-law, 20 male spouses, 22 CHVs and 16 key informants including medical officers, public health promotion officers, traditional birth attendants, reproductive health officers, a nutritionist, local chiefs and religious leaders. Data from one pregnant woman is not available for this study due to poor audio recordings.

The pregnant and postpartum women we interviewed were aged 17 to 39, had primary school education and the majority of them (over 60%) were unemployed, while a few participated in casual and informal work. Women under 18 years were considered emancipated minors if married. The mothers-in-law were aged between 35 and 63 years, the majority had no formal education, practised farming for economic activities and had relatively large families. The male spouses interviewed were aged between 24 and 63 years, were self-employed and had completed at least high school. Most CHVs had their own small businesses, worked between 10 and 30 h per week and had been in the role for more than two years. The Key informants had worked at least for two years in their respective ministries in a senior role. Tables 2, 3 and 4 (appendix 1) summarise our participants socio-demographics.

### Key findings: perceived effects of high temperatures on postpartum women and their neonates

Our findings from community members interviewed for this study suggest that high temperatures affect mothers and their neonates in multiple ways. Several health conditions in neonates were perceived to be a direct effect of the heat, including injuries on the skin and mouth, as well as impact on the wellbeing and behavior of the neonate (distress, crying). Heat impacts made it difficult to feed, interact and bath the neonate. In addition, the heat had significant effects on the postpartum women, including exhaustion, discomfort, and the inability for mothers to take care of themselves. These heat effects on the mother also have implications for infant care and wellbeing.

Heat impacts were exacerbated by the drought. The post-partum women reported increased workload because water sources were farther away, and negative impacts on agriculture and loss of shading around the home. This increased the women’s exhaustion in the heat. Community members noted that some postpartum women re-commenced household duties such as fetching water and firewood and farming immediately after birth. Our participants linked these chores with longer periods of postpartum bleeding. In addition, heat and insufficient water led to reduced personal hygiene. Houses are often poorly ventilated and participants reported that the indoor heat made it difficult to use mosquito nets, which would protect mother and baby from malaria. Many factors contributed to a reduced quality of care for the neonate, and these are explored in more detail below.

Low agricultural yields were associated with food scarcity and perceived to lead to low breast milk production, making it difficult for the mother to breastfeed exclusively. Additionally, mothers characterized both indoor and outdoor environments as inhospitable during periods of intense heat, reaching the point where they felt they had “nowhere to escape. We present a conceptual framework (described in supplementary Fig. 1) that summarizes the direct and indirect pathways of high temperatures on postpartum women and their newborns.

#### Heat-related skin problems in neonates

Participants described how they are beginning to see more neonates with damaged skin and attributed this to high temperatures becoming more common. Common descriptions of these injuries included “blisters’, “skin peeling off”, “heat rash”, and “skin patches”. Blisters were reported to develop immediately after birth, if not at birth, making it difficult to care for neonates.

The temperature was higher than usual [and] one week after delivering the baby had blisters … You couldn’t be able to undress the baby because those blisters were producing water, and in cases [where] they burst, they formed a wound because of the warm temperature.

IDI - Postpartum woman, Chalani.

Male spouses shared similar sentiments about blisters developing into wounds on the babies’ skin.

The baby is normally affected because of hot temperatures. The baby gets blister like wounds …because of hot temperatures. And in most cases, the baby cries very often because of the heat.

FGD- Male CHVs, Kasemene,

CHVs reported seeing babies developing blisters all over their bodies including the head.

Also, these babies who are born normally develop ‘*malenge lenge*’ (blisters; Kiswahili). You can get a baby who has developed those *malenge lenge* all over the body. Even up to the head. That is due to the heat. Even if it is born at full term, some of them are normally born when they have already developed those *malenge lenge*.

FGD - Female CHVs, Ribe.

Key informants and male spouses shared similar observations but described it as “heat rash”, “skin patches”, and “skin peeling off” rather than as “blisters”.

You will get people telling you that the baby has a fever, even if they [the babies] have a heat rash. What is worse is [that] they [babies] will be crying and scratching themselves, instead of breastfeeding.

KII - Ministry of Health Official, Kilifi.

… I even have experienced one of my children suffer[ing] skin rashes and something like the skin has some patches and when I consulted a clinician, definitely the answer was that this is heat rash, which is so extreme that even the child cannot sleep during the night, the child was crying and, in most cases, it is not treatable.

KII, Public Health Official- Kilifi.

The heat situation affects the baby’s skin. … You may find the baby has rashes, then the peeling off (skin) and crying a lot.

FGD, male spouses, Gotani.

A community chief who was a key informant for the study shared the same views and noted that local behavioral practices such as covering small babies with multiple layers of clothes may amplify the heat burden on the neonate’s already compromised skin.

It [heat] really affects [the baby] and you would find that the skin of the young baby [is] peeling off, the first skin peeling off because of the heat. And as [in] my community [they] say that the baby must be totally covered, … so it has to be totally covered and so we totally cover the baby, and it is very hot, then the skin peels off.

KII, Chief, Kilifi.

The chiefs’ reports aligned with field researchers’ observations that neonates were typically covered in many layers of clothes and/or blankets during the interviews.

#### Difficulties in caring for the neonate in the heat

The direct effect of heat makes babies uncomfortable, making it difficult for mothers to take care of them. Some male spouses described neonates as “having no peace”, “uncomfortable”, “crying all the time”, “cannot sleep”, or “can’t concentrate on breastfeeding”.

…the baby has no peace in the night, hot temperatures make the baby to cry, the baby is never comfortable at all.

Across our participants, there was a consensus on the difficulties postpartum women experienced taking care of their babies.

I would undress myself and the same to the baby; if the baby has blisters, you can’t even breastfeed him, instead, [baby] keeps on crying.

IDI, postpartum woman, Chalani.

Male spouses interviewed in this study also alluded to mothers’ difficulties breastfeeding their babies due to the discomfort caused by the heat.

The extreme heat affects the baby during breastfeeding, it affects the baby because she can’t concentrate on breastfeeding… even breastfeeding she can’t do it well. … the baby should be breastfed throughout, but when there is more heat, the baby is uncomfortable to breastfeed as he cries due to hot temperatures. It is really a problem.

FGD, Male Spouses, Viragoni.

Reports from community health volunteers and key informants from the ministry of health suggest that Kangaroo Mother Care (KMC), a method recommended to improve the wellbeing and development of low birthweight babies by maximising skin-to-skin contact, is challenging to perform in the condition of extreme heat. KMC was reported to increase perspiration in both mothers and babies, resulting in intense discomfort for both.

It will be a problem for the baby to be put in Kangaroo style. For you yourself [post-partum mother], you are already sweating all the time. So how will you put that baby into the Kangaroo style? Will you not be endangering the baby more? It will be trouble on top of trouble.

FGD, Female Community Health Volunteers, Rabai.

Views from some stakeholders align with observations made by CHVs -however, for some key informants like the community health officers, hot temperatures’ effect on water supply implies that mothers may not bathe daily, affecting their bodily hygiene. Thus, they did not view KMC as beneficial due to the lack of hygiene in many mothers.

…, it is a good idea [KMC] but with aspects of hygiene, something needs to be checked and adhered to. Because we could be advocating for Kangaroo for the mother but looking at the hygiene status, the probability of having water – like how many times does that mother take bath? It is also an issue that needs to be considered, so it all goes around the hygiene and the whole benefit of Kangaroo.

KII, Community health officer, Rabai.

- ***Contextual factors that increase heat exposures and affect the care of the neonate.***

Indoor heat in the poorly ventilated homes increased discomfort among the mother and the neonate making it cumbersome for the mother to breastfeed. Some participants said that the heat does not subside at night, which may harm both the mother and child.

When she [mother] is in that house, cooking is in there, everything in there, with a newborn baby, it is a must that it will affect the baby because of the smoke, dust, and hotness. Sometimes [baby] will be crying at night, and you wonder what the matter is, yet it is the house’s condition as it is because it lacks windows, nothing, no coldness, it’s hot. It disturbs the child….

FGD, Mothers-in-law, Buni.

Male spouses described how cooking with solid fuel in these homes intersects with high temperatures, and with there being not enough space to escape, causing sweating.

According to our living standards, you will find that as common citizens, we prepare food in a small house using firewood. The heat becomes too much inside the small houses [and] they start sweating. They wish to strip naked but cannot.

FGD, Male Spouses, Kamkomani.

These conditions inside homes were said to make it difficult to use mosquito nets. It was reported that mothers refused to use mosquito nets because they heightened the severity of the heat.

During hot periods, they fold up their mosquito nets and put them on the hung line because they say sleeping under them increases the already high heat. So, sleeping is like that [sleeping without mosquitoe net]. So, it affects women and even newborns. Therefore, it now affects almost everyone.

FGD - Male CHV-Kasemeni.

Further, our data suggests that mothers’ participation in household duties such as fetching water takes them away from their neonates for many hours. Participants had observed mothers getting stressed performing these chores and viewed this as hurting milk production.

…a postnatal woman has to walk about two kilometers searching for water. And maybe she must go with about two or three other people and if she has to make three or four trips, she gets very tired. And if she gets very tired, a neonatal baby won’t get enough breast milk because the mother is stressed up because of thinking about where and how to get water and other household essentials. And when they are stressed, breast milk production is not there.

FGD, Male Community Health Volunteers, Kilifi.

Spending many hours performing activities of daily living (ADL) outside the home was also linked to mothers decision to introduce solid food to their babies prematurely, i.e. earlier than 6 months as reported by a key informant from the ministry of health.

It affects the mother because—due to the tasks that she performs in the sun—she will not produce enough milk to breastfeed the child. Therefore, the child is introduced to weaning before six months elapse. They (nursing mothers) have no (option) otherwise, and they have to ensure the child is full by the time they go to the farm or walk long distances to collect water. The child is forced early to eat things it should not, due to circumstance, and this is a risk to it and the mother. I think people [in Kilifi] do not understand [the dangers]. They think that [introducing solid food early] is the best.

Participants described how some villages have not harvested for three years, while in other areas, participants report low yields and animal deaths owing to water scarcity. Moreover, the interplay between high ambient temperatures, drought and food insecurity was potentially linked to low breast milk production, which in turn was seen as influencing mother’s ability to perform exclusive breastfeeding.

The support is needed because this woman who has no food and she is exclusively breastfeeding—you will find they [the babies] don’t reach the [age of] 6 months. That is the truth! You will find a woman has started feeding the baby on food, aaaha! You will ask her why she is feeding this baby, and he hasn’t attained that level [of development]? She will answer you that ‘There is nothing here [no milk in the breast]. Nothing is coming out [milk is not coming out of the breast], and the baby is crying.’ Another woman will say it: ‘[My baby] keeps breastfeeding. … I have to look for porridge, and that porridge— remember—it will be without sugar. There is a challenge [here], there is a challenge with [lack of] food. That is the truth! If we want people to be supported, there is a problem with food. There are people who are suffering….

FGD, Female Community Health Volunteers, Ribe.

#### Exhaustion and delayed postnatal care

When asked how high temperatures affect postpartum women, community members reported that consequences for women included exhaustion and delayed postnatal care. Key informants for this study reported that the delay in post-natal attendance arises from the communal notion of the baby’s skin being delicate and not wanting to endanger the baby in the scorching sunshine.

There is a notion that is around, which is not a notion as such but a belief that the infant’s skin is a bit delicate and sensitive… so, if one thinks that ‘I have a few kilometers to get to the hospital for the post-natal clinic, then along the way I will be exposing my kid to the dust, direct heat and sun’ – which they feel is not good.

KII, Public Health Officer, Rabai.

It [high temperatures] reduces [post-natal attendance] because you can’t walk too far, most of our facilities are too far from the villages, so you can’t walk to the facility, so most of them [postpartum women] will not attend the clinics.

KII, Community Health Officer, Kaloleni.

Although distance to the health facility was described as one of the causes of a delay in or even reduced uptake of postnatal care, however, CHVs and community chiefs suggested that the fear of walking in the heat intersects with women’s exhaustion from other activities of daily living such as fetching water early in the morning.

They will prefer not to go to the hospital after walking all that distance for water. Therefore, they will not be able to follow up on their progress with the clinics.

KII, Chief, Kaloleni.

Sometime as hot as it is and if one is very far from the dispensary or the health facility and she is supposed to go, before visiting the facility she has to make sure there is availability of water inside the house…. So, she may delay and decide not to go after fetching the water, being very tired and it is a distance to the health facility, she may decide either to attend late or not to attend

KII, Chief, Rabai.

In periods of high temperatures, postpartum women may prioritize fetching water as opposed to attending postnatal clinics. The views of community health volunteers below align with the chiefs and other community members’ observations.

Women in our place, they really fear these hot temperatures…maybe someone woke up in the morning to go look for water. Because the most important item in the house is water. She has taken two hours to fetch water. When she comes back, the temperature is already gone up. It is really “burning” very much. Do you think she can carry the baby? And maybe she doesn’t have even an umbrella. She will say, she will go tomorrow, and tomorrow and like that, like that because water problem is a daily routine. And when she comes, the temperatures are already gone up. She postpones again.

FGD, Community Health Volunteer, Ribe.

#### Longer healing period for mothers

Male spouses and CHVs linked high ambient temperatures to prolonged postnatal bleeding and longer periods of healing in the postpartum

As for the mother, after delivery, she experiences unusual things, such as bleeding, she never gets dry fast.

FGD, male spouses, Kamokamani,

…The state of ability to heal will be there but it is slow. It won’t be that one which happens fast enough. It is because during hot temperature, the wound is normally watery, and this makes it not to heal fast.

FGD, Community Health Volunteers, Ribe.

Similar sentiments were shared by the key informants who also associated extreme heat with slow healing in postpartum.

It (high temperatures) can affect the healing for those who have C section … I think it can just affect the healing of the mother. Because of the wound, when you have a wound and when it is too hot, it doesn’t heal that fast.

KII, Community Health Officer, Kaloleni.

## Discussion

Diverse participants in this study described poor health outcomes in neonates in Kilifi and attributed skin problems, discomfort and restlessness, and a reduced nutritional intake during high ambient temperatures in the County. The discomfort was exacerbated by intolerable indoor home environments, making it challenging for mothers to provide care and for infants to breastfeed and sleep. Participants in the study believed that the insufficient breast milk production and the early introduction of alternative foods to infants before six months of age were attributed to the impact of high temperatures on food production. Additionally, they pointed out that the extended hours mothers spent performing outdoor chores away from home also contributed to these feeding practices. It remains uncertain whether all the health issues mentioned by participants can be definitively linked to the prevailing heat, however.

### Breastfeeding

A World Health Organization (WHO) and a United Nations Children and Environment Fund (UNICEF) Global Breastfeeding Scorecard Report shows that worldwide, 41% of children under the age of six months were exclusively breastfed in 2019 [32]. In Africa, more than 95% of infants aged 6 months are breastfed, but the rate of exclusive breastfeeding (EBF) is reported to be low, with exceptions in Rwanda and Burundi where EBF is reported to be > 80% up to age 6 months [33]. Under six months, the rate of EBF in Kenya has been estimated to be 61% [34]. Pre-lacteal feeding includes water and other liquids and is caused by factors such as maternal education, C-section, the sex of the child and home delivery [35, 36].

Community members in Kilifi identified low rates of EBF through three main mechanisms: (1) direct high ambient temperatures may cause blisters on the baby’s skin and in the mouth, which are uncomfortable for the baby; (2) low breast milk production linked to scarcity of food, and (3) raised temperatures in poorly ventilated houses exacerbating discomfort for both mothers and babies. These observed linkages require further scientific investigation.

Barriers to breastfeeding – including those related to heat – are interlinked, and interventions must address them from a systems perspective. Firstly, there is a need to provide cool comfortable spaces where women can nurse when it is hot, both in the communities and in health facilities, by equipping them with electricity for fans and cooling. Secondly, there is a need for programs and policies in settings such as Kilifi to provide the essentials, including supplies of clean water and nutritious foods for pregnant and nursing women, to overcome the problem of food and water insecurity for this vulnerable group. Thirdly, CHVs should be strategically utilized as conduits for disseminating education on the significance of breastfeeding during hot weather and the imperative of maintaining proper hydration. Furthermore, enhanced guidance from healthcare providers is key, as they play a critical role in informing and advising pregnant and postpartum women and their families on EBF, while also incorporating the active involvement of partners and fathers [37, 38]. Finally, substantial investments to ameliorate the built environment are requisite, necessitating multi-sectoral coordination and innovative methodologies to cool indoor living spaces, particularly in resource-constrained settings. Initiatives such as reducing indoor pollution through the provision of eco-friendly cooking stoves with lower emissions, heightened fuel efficiency, and improved air quality to mothers in these locales may further contribute to mitigating the perceived temperature in homes.

### Direct effect on the skin

Although skin diseases are common in children in Africa [39, 40], published research in this area is scant, particularly skin diseases in neonates. In our study, the impacts of heat were perceived to cause a range of skin conditions in infants. Although clinically, there are skin conditions caused by heat (e.g. prickly heat), it is difficult to interpret which skin conditions were more prevalent and the role of heat or sunlight. Researchers in this study observed the common practice of heavily layering neonates, attributed to cultural beliefs aimed at protecting them from the ‘evil eye.’ These practices potentially exacerbate the heat burden on neonatal skin. Addressing these customs requires an awareness-raising and behavior change intervention, leveraging key influencers such as chiefs, Community Health Volunteers (CHVs), community elders, and public health officers. Furthermore, to provide appropriate care for infant skin, there is a need for clinical research focusing on skin diseases in newborns in the region. Such research could elucidate the correlation between high ambient temperatures and skin diseases in neonates.

### Kangaroo Mother Care

Skin-to-skin care is protective against a wide variety of adverse neonatal outcomes, especially in those born too soon, and facilitates close observation of the baby. KMC has the potential to prevent many neonatal complications and even improves exclusive breastfeeding. However, swaddling neonates when it is hot can be harmful because of the baby’s poor thermoregulatory capacity as they cannot dispose of the excess heat [41, 42]. Sharing a bed with a newborn during periods of extreme high temperature is not recommended as it may result in child death [43]. Findings from this study suggest that mothers may be reluctant to practice KMC in the heat, owing to the discomfort involved. The need to improve community health facilities for KMC is key, considering that many Kilifian mothers give birth to underweight babies [44, 45]. Interventions such as solar-powered electricity to cool buildings and health facilities are warranted.

### Delayed postnatal care and slower healing processes

Engaging in household and outdoor chores under elevated temperatures was reported to precipitate exhaustion among mothers. It is imperative to initiate community-wide educational campaigns elucidating the perils associated with climate change and the impact of heightened temperatures on postpartum women and neonates. Such awareness initiatives are pivotal in fostering community support mechanisms, particularly in alleviating the burden of daily chores on women in these communities. Furthermore, strategic investments are warranted in establishing healthcare facilities proximate to residences, ensuring enhanced accessibility for women seeking postnatal care (PNC) during hot weather conditions. Simultaneously, considerations for bolstering transportation infrastructure to existing facilities within Kilifi County are crucial components of a comprehensive approach to maternal and neonatal well-being.

### Priority interventions

Delivery of heat-health early warning weather information to inform mothers to take protective measures when it is hot is urgently needed. CHVs who work closely with women in the communities could be trained to deliver these messages. Community education on extreme heat impacts on mothers and their newborns health and wellbeing is urgently required. Importantly, greening the homes and health facilities and ensuring that women have accessible clean water in their homes and health facilities is an essential first step towards alleviating the effects of heat exposure.

### Strengths and limitations

Being the first study of its kind in East Africa, particularly in a region that experiences high temperatures and drought throughout the year, these findings contribute to a much-neglected area. Our study participants were carefully selected to include community members who could speak directly about their observed experiences of the effects of high temperatures on women and their neonates. The study’s qualitative and anthropological approach to data collection enabled us to build a nuanced understanding of how extreme heat impacts neonatal health. The intersection between drought and food insecurity was a significant and dominating concern. Drought clearly exacerbates the heat risks, thus highlighting the importance of also looking at women’s experiences in non-drought situations. Dermatology was an unexpected finding for researchers, and therefore we have limited data to understand what exactly the described skin diseases are. There were also missed opportunities in the interviewing where themes such as dehydration and interactions between mother and baby and the help infants received while mothers were away were not adequately followed up and explored. Nonetheless, interviewing a wide range of community members who support women as they welcome their neonates into the world provided deep insights into their experience. Lastly, it is important to highlight that our qualitative data is based on community members views, and epidemiological evidence would help to establish an association between neonatal outcomes and high temperatures. Data from the Kilifi Health Demographic Surveillance System (KHDSS) for Young Infants (YI), described as children below 60 days, indicate high mortality rates due several factors, including low birth weight, sepsis, birth asphyxia and preterm complications [45].

## Conclusions

High ambient temperatures impact directly on neonatal health, bringing discomfort and skin problems while reducing women’s ability to exclusively breastfeed and undertake other aspects of infant care. Heat exposures are exacerbated by poor housing, in which indoor temperatures are raised by indoor fires and inadequately ventilated homes. High temperatures and drought increase the physical burden upon women by increasing the distances that need to be covered in the heat, and the drought has increased malnutrition in mothers and babies. These findings point to the need to initiate and implement multi-sectoral policies and programs to mitigate the negative impacts of extreme heat on maternal and neonatal health in rural Kilifi and similar settings. As the earth continues to warm over the coming decades, such interventions will become essential. Our finding shows the emerging gaps that need to be addressed to maintain the progress that has been made in the last decade in reducing the maternal and neonatal mortalities. Climate change is a threat to neonatal health and there is an urgent need to put strategies and interventions in place to support neonatal care in low-and-middle-income countries.

### Electronic supplementary material

Below is the link to the electronic supplementary material.

Supplementary Material 1

Supplementary Material 2

Supplementary Material 3

Supplementary Material 4

## Data Availability

The datasets generated and/or analyzed during the current study are not publicly available because it is possible for someone from the study sites to deduce participants if they get access to the full transcript but are available from the corresponding author on reasonable request.
