# Supplementary material for: Too hot to thrive: a qualitative inquiry of community perspectives on the effect of high ambient temperature on postpartum women and neonates in Kilifi, Kenya
Source: BMC Pediatr. 2024 Jan 13;24:36. doi: 10.1186/s12887-023-04517-w (PMC10787431; doi:10.1186/s12887-023-04517-w)
Supplement: Supplementary file 1 — Supplementary Material 1 [file 12887_2023_4517_MOESM1_ESM.docx]

**APPENDIX 1: TABLES**

**TABLE 1: MONTHLY WEATHER SUMMARY IN KILIFI**

**TABLE 2: SOCIO DEMOGRAPHICS FOR PREGNANT AND POSTPARTUM WOMEN**

**** We excluded data for one pregnant woman due to poor audio recordings***.**

**TABLE 3: SOCIO DEMOGRAPHICS FOR THE FOCUS GROUP DISCUSSIONS**

**TABLE 4: A SUMMARY OF KEY INFORMANT INTERVIEWS**

|  | **Role** | **Years in Role** |
| --- | --- | --- |
| 1 | Community public health promotion officer | 20 |
| 2 | Public health promotion specialist | 8 |
| 3 | Public health officer | 10 |
| 4 | Community health officer | 6 |
| 5 | Nutritionist | 12 |
| 6 | Senior chief | 15 |
| 7 | Medical officer | 7 |
| 8 | Reproductive health specialist | 8 |
| 9 | Traditional Birth Attendant -1 | 10 |
| 10 | Assistant Chief | 5 |
| 11 | Religious leader | 10 |
| 12 | Environmental officer | 6 |
| 13 | County reproductive health specialist | 24 |
| 14 | Extension officer | 3 |
| 15 | Environmental manager | 5 |
| 16 | Traditional Birth Attendants -2 | 10 |
