# Supplementary material for: Too hot to thrive: a qualitative inquiry of community perspectives on the effect of high ambient temperature on postpartum women and neonates in Kilifi, Kenya
Source: BMC Pediatr. 2024 Jan 13;24:36. doi: 10.1186/s12887-023-04517-w (PMC10787431; doi:10.1186/s12887-023-04517-w)
Supplement: Supplementary file 3 — Supplementary Material 3 [file 12887_2023_4517_MOESM3_ESM.docx]

# **APPENDIX 3: RESEARCH QUESTIONS**

# Topic Guide, IDIs with Postpartum Women

| **Key Question posed to participant** | **Probes/ Subsidiary Questions** |
| --- | --- |
| **Experience and perceptions of extreme heat in late pregnancy and at childbirth** |  |
| 1. Please tell me about the final weeks of your pregnancy. | Probe:   - Health and wellbeing - Expectations to continue working. - Support from other household members - Visits to health facilities |
| 1. Were there periods of very hot weather at any point during that time? | - Tell me more, what do you remember about this experience? - How did the heat affect you? - How did the heat affect your baby? |
| 1. Tell me about the birth. | - If at home: why did you choose to give birth at home? - If at facility: why did you choose to give birth at the facility? - How did you get to the facility? - Duration of labour - Involvement of healthcare workers, family members - Health and wellbeing of baby at birth |
| 1. What is your memory of the temperatures indoors and outdoors when you were in labour, during childbirth and immediately afterwards? |  |
| 1. If you experienced extreme heat around the time of giving birth, how did this affect you? | Probe – impact on:   - Decision to get to the birthing facility (if relevant) - Ability to get to the birthing facility and be well-prepared for the birth (e.g. source own water) - Experience of giving birth - Ability to endure labour - More thirsty? - Length of labour - Chance of bleeding - Ability to recover from childbirth (first few hours) - Other? |
| 1. How did the extreme heat around the time of birth affect your baby’s health & wellbeing? | Probe:   - Bonding between mother and baby immediately after birth - Breastfeeding soon after birth - Listlessness |
| **Coping strategies when giving birth** |  |
| What did you do to cope with the heat (for yourself and for your baby) around the time of birth? | Probe for examples:   - Drink more water or other cold fluids - Wash/shower with cold water - Use a fan in the home - Put feet in the soil/ cold water - Sit under trees |
| How successful were these measures? |  |
| Who else offered support to you to minimize the effect of heat around the time of birth? | E.g., mothers-in-law, TBAs, CHVs, spouse, HCW |
| What forms did this support take? |  |
| How much did this support help? | What could have been done better? |
| 1. Are there other things that could be done within communities to help women cope better with or escape extreme heat when they are giving birth? | Could be specific interventions or forms of support |
| 1. What are the possible barriers to implementing the things you have just mentioned? Enabling factors? |  |
| 1. Are you aware of any measures that the health ministry have put in place to try to support mothers and babies during and after delivery when it is very hot? | If any, how successful do you think they are? |
| **Experience of extreme heat in postpartum period and coping strategies** |  |
| 1. Since you delivered your baby, where do you spend most of your time? | E.g. in your house, outdoors, with friends, at the church/ mosque, etc? |
| 1. What is the temperature like in these places? | How are your choices of where you spend your time since giving birth influenced by the heat, if at all? |
| 1. What is your experience of the temperatures **inside** your home? Is this different from the temperatures **outside** the house? | If different/or not, please explain |
| 1. If there have been very hot days since giving birth, how has your baby been affected by this? | - Impact on health and wellbeing - Behavioural changes (e.g. sleep patterns, excess sweating and crying, rashes, dehydration...) - Baby unwell and needed medical attention? |
| 1. What have you done to help your baby during very hot weather? | - How do you keep the baby hydrated? - Is there anything you have done differently (compared to in cooler weather)? E.g. bathe the baby, breastfeed less, give water to the baby, etc. - How successful have these measures been in cooling the baby down or keeping her/him hydrated? |
| 1. What have other people in your household done to help you and your baby cope with the heat since the birth? | How helpful are these actions for you and your baby? |
| 1. Have you received any health information about strategies to cope with heat stress when you have a new baby? | - Source of information - Content - Utility: has this information been helpful in controlling heat stress for you and your child? How is it helpful or not? |
| In your view, what polices/strategies do you think should be implemented to help women and their newborn babies in communal spaces? | - Why these policies/strategies? - Who should be responsible for implementing them? |
| **Weather forecasting & resilience to heat** |  |
| 1. Do you ever search for information that predicts what weather to expect? | Probe:   - From where/whom? (including community sources, e.g. a rainmaker) - In what form? - How often? - What time of the year? - Helpful/not helpful? |
| 1. How does this information change your behaviour or day-to-day planning, if at all? | - What would you do in the home to prepare for a predicted heatwave? |
| 1. How could weather forecasting help you and other women with new babies to prepare better for very hot weather? | - What kind of information would be most useful? - What are the best ways of communicating this information? |

# Topic Guide, KIIs with Traditional Birth Attendants (TBAs)

| **Key Question posed to participant** | **Probes/ Subsidiary Questions** |
| --- | --- |
| **Experience and perceptions of extreme heat in late pregnancy and at childbirth** |  |
| 1. How did you come to be a TBA? |  |
| 1. What is the size of the population you cover? How many women do you deliver in a week? month? Year? |  |
| 1. Could you tell me a little bit more about your role assisting pregnant women, from the early stages of their pregnancy to delivery. | Probe:   - How do you get your clients? - What help/assistance do you give them during pregnancy? - Are there cultural practices you follow? - What about if the woman is high risk pregnancy –what do you do during complications? - Where do you deliver them and why? - How much do you interact with HCWs in the clinics/hospital? And with CHVs? |
| 1. When are the very hot periods in this area? | - Probe about heat in dry versus wet seasons - How has the weather in this area changed, if at all, in recent years? (e.g. hotter summers, spring heatwaves, droughts, flooding) - What do you think is causing these changes? |
| 1. How is the supply of power and water in the community affected during periods of very hot weather? | - How does this affect the delivery of services to women during pregnancy and at actual delivery? |
| 1. Do you notice a change in the volume and type of complaints being presented by women during very hot weather? |  |
| 1. How does the hot weather affect the care you provide to women when they are pregnant and when they give birth? | Probe about:   - Managing dehydration - Monitoring during labour and delivery - Postpartum observation - Decision-making about birth place - Referrals in high risk cases |
| **Views on how women and neonates are affected by heat** |  |
| 1. In your view, how does very hot weather affect women during pregnancy? | Probe – impact on:   - Ability to rest/sleep - Ability to work - Mobility - Eating patterns - Ability to secure food, water and fuel for cooking - Ability or willingness to access healthcare services - Behavioural changes (e.g. more irritable, lethargic, less energetic) - Relationships at household and community levels |
| 1. In your experience, are pregnant women any more or less sensitive to heat than women who are not pregnant? |  |
| 1. How do you think high temperatures affect the baby in the womb, if at all? | Probe – impact on:   - Growth and development of foetus - Movement of foetus in the womb - Does heat stress make the baby come earlier, at the normal time or after the normal time? - Other? |
| 1. How does very hot weather affect women during labour and delivery? | Probe:   - More thirsty? - Length of labour: shorter, the same or longer - Chance of bleeding, more pain - Choice of birthing venue |
| 1. How does very hot weather affect the newborn baby? | Probe:   - Recovery from birth - APGAR scores - Ability to feed (latch on) - Need for extra hydration - What else? |
| 1. How do high temperatures affect women in the first few weeks after giving birth? | Probe:   - Breastfeeding - Kangaroo care - Recovery from pregnancy |
| 1. How do high temperatures affect young babies (up to 6 weeks old)? | Probe – impact on:   - Crying - Thirst or dehydration - Breastfeeding e.g. the number of times and length of time they breastfeed - Sweating - Fever - Movement (e.g. more still on hot days?) - Bonding with the baby’s mother - Growth or development |
| 1. Are there illnesses that are more common in young babies when the temperatures are very high? |  |
| 1. How does heat compare to other threats to health in pregnancy and young babies, in terms of severity and outcome? | E.g. compare to smoking or drinking alcohol during pregnancy? |
| **Heat Adaptation** |  |
| 1. What advice do you give the women on how to minimise the impact of hot weather? |  |
| 1. Is minimising heat stress among pregnant women and newborn babies during delivery a priority for you? | Why/ why not? |
| 1. What do you think should be done in the communities to help pregnant women and newborn babies during very hot weather? | Examples:   - Provide sustainable access to water, encourage more drinking - Improve built environment, ventilation, etc - Provide fans - Advise women on clothing removal - Use of wet cloths - Plant trees - Access to timely information - Built cool refugee spaces in the communities |
| 1. Who should be responsible for implementing these suggestions and why? | Examples.   - Ministry of health - Local leaderships - Religious leaders - Mothers-in-law - CHVs |
| 1. What is needed to implement the interventions you have suggested? |  |
| 1. What are the barriers to implementing these actions (i.e. to help pregnant women and neonates when it is hot)? |  |
| 1. What challenges do you face during hot weather in your work and how do you mitigate these challenges? |  |
| 1. What support do you need to do your work effectively during hot weather? | Who should provide this support? Why this person? |
| **Weather forecasting & resilience to heat** |  |
| 1. What kind of information do pregnant and postpartum women need about avoiding heat stress? | What are the best ways to communicate this info to them? |
| 1. Do you ever receive weather forecasts about extreme heat? | - For a short term period (0-5 days in advance) - For a longer term period (1-3 months in advance) - What do you do in response? |
| 1. What policies are you aware of that could help to safeguard the health of pregnant women and babies during very hot weather? | What do the policies say? |
| 1. What are the barriers to implementation of these policies? |  |
| 1. Looking forward, what further support / tools / information do you think would make the biggest difference to improving resilience to hot weather events in the community? |  |

Topic Guide, KIIs with Community Representatives [e.g. CHC members, village leaders, religious leaders and community public health officials]

| **Key question asked of the participant** | **Probes / Subsidiary Questions** |
| --- | --- |
| **Community environment** |  |
| 1. Please tell me more about your role and responsibility within the community | - How did you come to occupy this role? - How long you have been in this role? |
| 1. How much do you interact with pregnant women, new mothers and their babies, in this role? | How? |
| 1. When are the very hot periods in this area? | - Probe about heat during dry and humid seasons - How has the weather in this region changed, if at all, in recent years? (For example, warmer dry seasons, winter heat waves, droughts, floods) - What do you think is causing these changes? |
| 1. How is the supply of electricity and water affected during periods of very hot weather? |  |
| 1. What is the effect on people’s bodies when the weather is very hot? | - What changes (physical, psychological) have you noticed in people when temperatures are very high? - Are there more common diseases when temperatures are very high? |
| **Opinions on how women and newborns are affected by heat** |  |
| 1. How do you think very hot weather affects women during pregnancy? | Probe - impact on:   - Ability to rest/sleep - Work capacity - Mobility - Eating habits - Ability to secure food, water and fuel for cooking - Ability or willingness to access health services - Behavioural changes (e.g. more irritable, lethargic, less energetic) - Household and community relations |
| 1. How do you think very hot weather affects the baby in the womb, if any? | Probe - impact on:   - Fetal growth and development - Movement of the fetus in the womb - Does heat stress cause the baby to come earlier, at normal time or after normal time? - Other? |
| 1. How does very hot weather affect women during labour and childbirth? | Probe:   - More thirsty? - Working time: shorter, identical or longer - Risk of bleeding, pain - Choice of place of birth |
| 1. How does very hot weather affect women in the first few weeks after giving birth? | Probe:   - Breastfeeding - Kangaroo Care - Recovery from pregnancy |
| 1. How do high temperatures affect newborn babies (up to 6 weeks)? | Probe - impact on:   - Crying - Breastfeeding, for example the number of times and duration of breastfeeding - Thirst or dehydration - Sweating - Fever - Movements (for example, more still on hot days?) - Bonding with the baby's mother - Growth or development   Are there illnesses that are more common in young babies when temperatures are very high? |
| 1. How does heat stress compare to other health threats during pregnancy and in young babies in terms of severity and outcomes? | For example, comparing with smoking or drinking alcohol or eating the wrong foods during pregnancy?  In babies – comparing with malnutrition? |
| **Heat adaptation** |  |
| 1. What cultural practices should women observe after childbirth? | Probe – relating to:   - Recovery from giving birth - Breastfeeding - Working in the home   How do these practices change, if at all, during periods of very hot weather? |
| 1. What is currently being done at community level to reduce the effects of heat stress among pregnant women, women giving birth, and newborn babies? |  |
| 1. How do you think members of the community can help improve the comfort of pregnant women and babies when it's very hot? | Examples?   - Advise women to rest more, open windows, use wet cloths, drink more water, etc - Fetch or carry water for pregnant women - Encourage spouses and those around them to help pregnant women - Help women to reduce their workload |
| 1. What can be done within the homes to minimise the impact of heat on women, either during pregnancy or when giving birth at home? | Examples...   - Make the home better ventilated - Plant more trees around the house - Improve access to water - Open the windows - Turn on the fans |
| 1. What can be done in communal spaces within the community to minimise heat impact for pregnant and postpartum women and their babies? |  |
| 1. What are the possible barriers to implement these suggested strategies? | What kind of support is needed to make this action possible? |
| 1. To what extent is this a priority for you? | A priority for community members? |
| 1. What policies do you know of that seek to protect the health of pregnant women and babies from heat waves and very high temperatures? | What do politicians say? |
| 1. What are the obstacles to the implementation of these policies in the communities? |  |
| 1. [For public health officers] To your knowledge, have there been discussions between Ministry of Health and community representatives about improving conditions in the homes during very hot weather? |  |

Topic Guide, Focus Group Discussions , Mothers-In-laws

| **Key Question posed to participant** | **Probes/ Subsidiary Questions** |
| --- | --- |
| **Experience and perceptions of extreme heat in pregnancy and at childbirth** |  |
| 1. What is the period in which very hot temperatures occur in this area? | How has the weather in this area changed, if at all, in recent years? (e.g. hotter summers, spring heatwaves, droughts, flooding)  What do you think is causing these changes? |
|  |  |
| 2. How do high temperatures affect pregnant women? | Probe – impact on:   - Ability to rest/sleep - Ability to work - Mobility - Eating patterns - Ability to secure food, water and fuel for cooking - Ability or willingness to access healthcare services - Behavioural changes (e.g. more irritable, lethargic, less energetic) - Relationships at household and community levels |
| 3. How do you think high temperatures affect the baby while it is still inside the womb, if at all? | Probe – impact on:   - Growth and development of foetus - Movement of foetus in the womb - Does heat stress make the baby come earlier, at the normal time or after the normal time? - Other? |
| 4. What is the temperature like indoors at health facilities in this area where women give birth? | Is it hotter/colder/same temperature compared to outdoors?  Do mothers-in-law normally accompany the women when they seek antenatal services or go to give birth? IF so, what is your role in these visits? |
| 5. How do high temperatures affect women at the time they are in labour and giving birth? | Probe:   - More thirsty? - Length of labour: shorter, the same or longer - Chance of bleeding - Choice of birthing venue |
| 6. How does heat compare to other threats to health in pregnancy and young babies, in terms of severity and outcome? | E.g. compare to smoking or drinking alcohol during pregnancy? |
| **Coping Strategies – pregnancy and childbirth** |  |
| 7. What are your roles as mothers-in-law during a woman’s pregnancy and childbirth? |  |
| 8. What is currently done in your homes to help pregnant women cope with high temperatures? | Are pregnant women able to reduce their physical workload in the home when it is very hot? |
| 9. How effective are the measures you have mentioned in reducing the effect of heat on pregnancy and child birth? | Some examples when the measures were effective? |
| 10. What could be done to help pregnant women cope with high temperatures in their homes? | Probe for examples:   - Rest more - Drink more water - Install ventilation |
| 11. What measures do women who attend ANC services at the facility say are used by HCWs to minimize the effect of heat? | Examples.   - Provide access to water - Open windows - Switch on fans |
| 12. During home births, what do TBAs do to help pregnant women when the weather is very hot? | Examples:   - Encourage women to sit on the banana leaves or/and shelter in the banana plantations? - Soak their feet in water? - Move women to a mosque/church? |
| 13. What could the community health volunteers do to minimize the effects of high temperatures on pregnant women and young infants? | - Are there other ways to help pregnant women cope better with or escape extreme heat? - What more could CHC members and TBAs do, specifically? - Need for information regarding heat stress? Referrals? |
| 14. As mothers-in-law, what do you do to help pregnant when the weather is very hot? | What would you do to help pregnant women if you had more resources available (money or building materials etc.)? |
| **Neonates and postpartum women** |  |
| 15. What are the cultural practices that women must observe after giving birth? | How are these adjusted (if at all) when it is very hot? |
| 16. As mothers-in-law, what are your roles in relation to the new mother and baby in your home? |  |
| 17. How do high temperatures affect women in the first few weeks after giving birth? | Probe:   - Breastfeeding - Kangaroo care - Recovery from pregnancy |
| 18. How do high temperatures affect young babies (up to 6 weeks old)? | Probe – impact on:   - Crying - Breastfeeding e.g. the number of times and length of time they breastfeed - Thirst or dehydration - Sweating - Fever - Movement (e.g. more still on hot days?) - Bonding with the baby’s mother - Growth or development - Are there illnesses that are more common in young babies when the temperatures are very high? |
| 19. What is currently done in your homes to protect young babies (up to 6 weeks old) when it is very hot? | In your opinion, how successful do you think these measures or coping strategies are? |
| 20. As mothers-in-law, what do you do for young babies when the weather is very hot? | What else would you do to help them if you had more resources available (money or building materials etc.)? |
| **Weather forecasting & resilience to heat** |  |
| 21. Do you ever search for information that predicts what weather to expect? | Probe:   - From where/whom? (including community sources, e.g. a rainmaker) - In what form? - How often and over what period? |
| 22. How helpful/unhelpful do you find this information to be? |  |
| 23. How does this information change your behaviour or day-to-day planning, if at all? | Especially when extreme heat is predicted |
| 24. If there was a warning that a heat wave was coming in the next few days, what could you do to help prepare a pregnant woman or woman and her new baby? |  |
