# Supplementary material for: Too hot to thrive: a qualitative inquiry of community perspectives on the effect of high ambient temperature on postpartum women and neonates in Kilifi, Kenya
Source: BMC Pediatr. 2024 Jan 13;24:36. doi: 10.1186/s12887-023-04517-w (PMC10787431; doi:10.1186/s12887-023-04517-w)
Supplement: Supplementary file 4 — Supplementary Material 4 [file 12887_2023_4517_MOESM4_ESM.docx]

**APPENDIX 4: SUMMARY OF THE STUDY CONCEPTUAL FRAMEWORK**


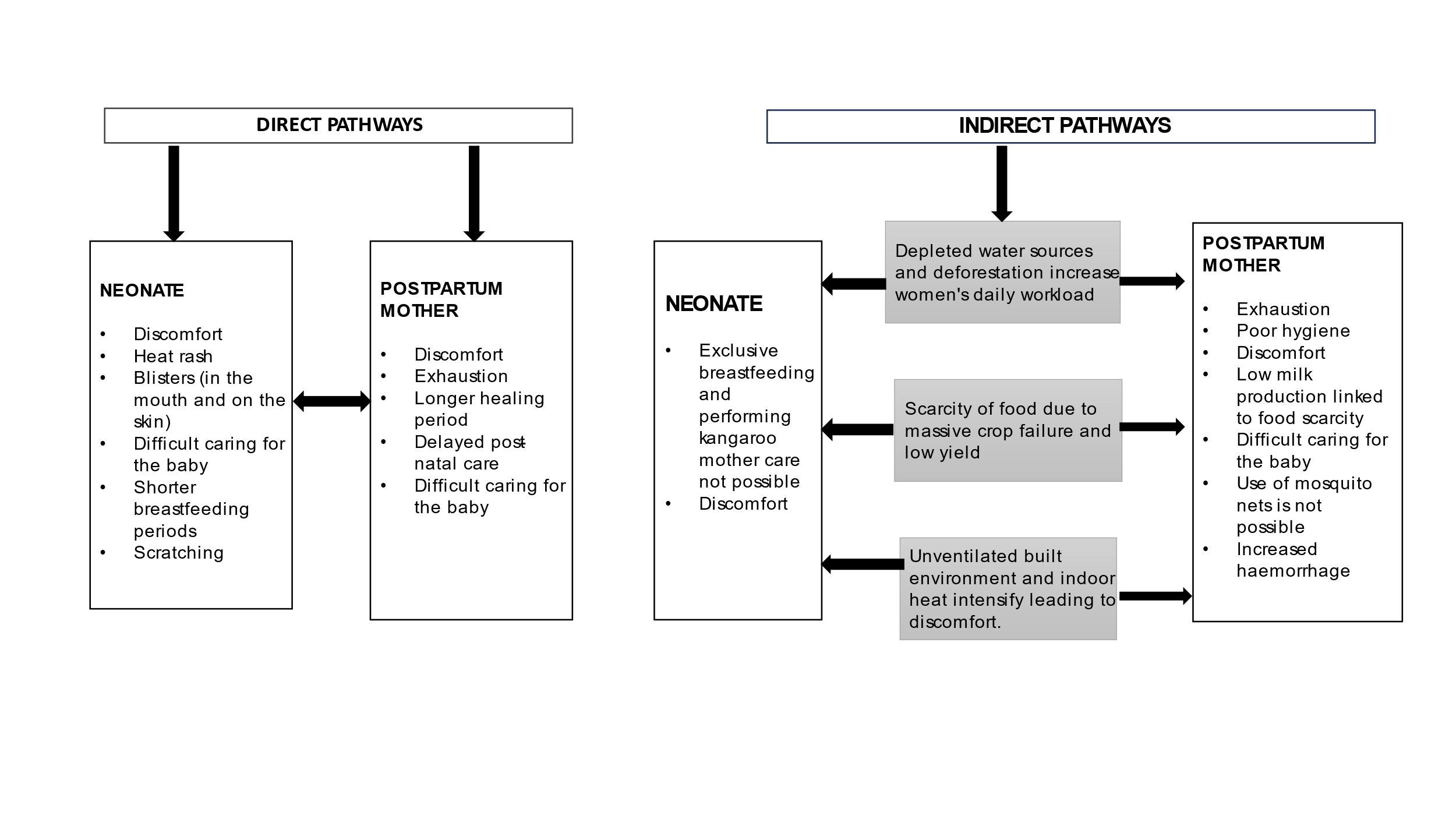
FIGURE 1: DIRECT AND INDIRECT PATHWAYS OF HEAT EXPOSURE ON POSTPARTUM WOMEN AND THEIR NEONATES
